# Hypertension and Exposure to Noise near Airports (HYENA): Study Design and Noise Exposure Assessment

**DOI:** 10.1289/ehp.8037

**Published:** 2005-07-13

**Authors:** Lars Jarup, Marie-Louise Dudley, Wolfgang Babisch, Danny Houthuijs, Wim Swart, Göran Pershagen, Gösta Bluhm, Klea Katsouyanni, Manolis Velonakis, Ennio Cadum, Federica Vigna-Taglianti

**Affiliations:** 1 Department of Epidemiology and Public Health, Imperial College London, London, United Kingdom; 2 Department of Environmental Hygiene, Federal Environmental Agency, Berlin, Germany; 3 National Institute for Public Health and the Environment, Bilthoven, the Netherlands; 4 Institute of Environmental Medicine, Karolinska Institutet, Stockholm, Sweden; 5 Department of Hygiene and Epidemiology, Medical School; 6 Laboratory of Prevention, Nurses School, University of Athens, Athens, Greece; 7 Environmental Epidemiologic Unit, Regional Agency for Environmental Protection, Piedmont Region, Grugliasco, Italy

**Keywords:** air pollution, aircraft, blood pressure, hypertension, noise, road traffic

## Abstract

An increasing number of people live near airports with considerable noise and air pollution. The Hypertension and Exposure to Noise near Airports (HYENA) project aims to assess the impact of airport-related noise exposure on blood pressure (BP) and cardiovascular disease using a cross-sectional study design. We selected 6,000 persons (45–70 years of age) who had lived at least 5 years near one of six major European airports. We used modeled aircraft noise contours, aiming to maximize exposure contrast. Automated BP instruments are used to reduce observer error. We designed a standardized questionnaire to collect data on annoyance, noise disturbance, and major confounders. Cortisol in saliva was collected in a subsample of the study population (*n* = 500) stratified by noise exposure level. To investigate short-term noise effects on BP and possible effects on nighttime BP dipping, we measured 24-hr BP and assessed continuous night noise in another sub-sample (*n* = 200). To ensure comparability between countries, we used common noise models to assess individual noise exposure, with a resolution of 1 dB(A). Modifiers of individual exposure, such as the orientation of living and bedroom toward roads, window-opening habits, and sound insulation, were assessed by the questionnaire. For four airports, we estimated exposure to air pollution to explore modifying effects of air pollution on cardiovascular disease. The project assesses exposure to traffic-related air pollutants, primarily using data from another project funded by the European Union (APMoSPHERE, Air Pollution Modelling for Support to Policy on Health and Environmental Risks in Europe).

An increasing number of people live in the vicinity of major airports and experience considerable noise and air pollution. Raised blood pressure (BP) is a major risk factor for coronary heart disease and the major risk factor for stroke (Whitworth 2003). Environmental noise is a significant problem in Europe, and it is estimated that roughly 20% of the European Union’s population (close to 80 million people) are exposed to noise levels that are considered unacceptable (European Commission 1996).

Few investigators have studied health effects associated with exposure to aircraft noise. Cardiovascular effects due to noise exposure have been studied to some extent, but no clear exposure–response relations are currently known (Babisch 2000), although a recent German study showed an excess risk of myocardial infarction related to traffic noise, but only in men (Babisch et al. 2005). An early European study showed higher treatment rates for “heart trouble” and hypertension among residents close to a major airport than among people living farther away (Knipschild 1977), and a later review found that hypertension was more prevalent among individuals living close to airports (Vacheron 1992). However, results are equivocal both with respect to BP increases (Babisch et al. 1990; Lercher et al. 2000; Pulles et al. 1990) and the prevalence of hypertension (Bluhm et al. 2001; Eiff and Neus 1980; Herbold et al. 1989; Knipschild and Sallé 1979; Maschke 2003). A recent cross-sectional study indicated an exposure–response relation between residence distance from a Swedish airport and hypertension (Rosenlund et al. 2001). Similar results were found in a community sample around a military airbase on Okinawa, Japan, and in a cross-sectional survey around Schiphol airport in Amsterdam, the Netherlands (Franssen et al. 2004; Matsui et al. 2004).

In 1999, the World Health Organization stated that the overall evidence available at the time suggested a weak association between long-term noise exposure and BP elevation or hypertension, and that cardiovascular effects are associated with long-term exposure to A-weighted average sound pressure levels [dB(A)] (*L*_Aeq,24hr_) throughout the day/night in the range of 65–70 dB(A) (Berglund et al. 1999). However, a recent German study suggested that traffic noise at lower levels might increase the risk of myocardial infarction and high BP, finding an increased odds ratio for medical treatment of hypertension in subjects with an exposure during the day/night of > 60/50 dB(A) compared with subjects with an exposure < 60/50 dB(A) (Maschke 2003). Recent studies suggest that nighttime exposure might be particularly relevant for health (Babisch et al. 1999; Health Council of the Netherlands 2004; Lercher and Kofler 1993; Maschke 2003).

Stress hormones are useful indicators to study mechanisms and interactions between noise and health outcomes such as BP (Babisch et al. 2001). The cortisol level is a good indicator of stress (Wust et al. 2000). Salivary cortisol correlates well with free levels of cortisol in serum, and correctly collected saliva samples have the advantage of being stable for long periods at room temperature (Hofman 2001), which facilitates their use in multicenter studies.

Community noise studies have traditionally considered only noise from a single specific source such as aircraft or road traffic. However, recent studies suggest that aircraft noise might be more annoying than road traffic noise (Miedema and Oudshoorn 2001), but the extent to which the findings from individual studies can be extrapolated to other environments is at present unclear. It is not meaningful to use a total integrated estimate of noise in studies of nonauditory effects, because sound energy is not the only factor that causes stress reactions. Attitudes toward noise and the activities disturbed by it may modify the effect of noise quite considerably, as well as objective characteristics such as time pattern and sound frequency distribution. However, in studies of effects of community noise, nuisance from other noise sources should be addressed as independent (or interacting) factors (as is commonly done in environmental epidemiologic studies) (Babisch 2002; Babisch et al. 2003; Pulles et al. 1990).

The literature relating hypertension to air pollution is sparse, and two recently published studies show contradictory results (Ibald-Mulli et al. 2004; Zanobetti et al. 2004), but there is a wealth of literature on air-pollution–related cardiovascular effects, particularly associated with short-term changes in particulate air pollution levels (Dockery 2001). A recent study showed increased cardiopulmonary mortality associated with living near major roads (Hoek et al. 2002). However, although mortality was associated with living near roads, there was less consistency in the relation with ambient air pollution concentrations. In spite of this, no attempts were made to adjust for road-traffic–related noise. Thus, it may be important to assess ambient air pollution exposure as a possible confounder/effect modifier of the association between community noise and cardiovascular risk.

The aim of the Hypertension and Exposure to Noise near Airports (HYENA) project is to assess the impacts on cardiovascular health (primarily reflected by high BP) of noise generated by aircraft and road traffic near six major European airports (Athens, Greece; Milano/ Malpensa, Italy; Amsterdam/Schiphol, the Netherlands; Stockholm/Arlanda, Sweden; Berlin/Tegel, Germany; and London/ Heathrow, UK). The project will identify and quantify noise exposure in individuals, relating exposure to prevalence of high BP.

The project will study a subsample of subjects in more detail, recording 24-hr BP (every 15 min) and continuous night noise measurements, to assess the short-term effects of aircraft noise during nighttime and its effects on BP nighttime dipping, which is an established risk factor for cardiovascular disease (Lee et al. 2005; Ohkubo et al. 2002).

The project will evaluate the modifying effects of traffic-related air pollution [nitrogen dioxide, particulate matter with aerodynamic diameter ≤10 μm (PM_10_)] on noise-associated cardiovascular risk factors and cardiovascular disease (high BP, ischemic heart disease). Standardized methods for assessing exposure and effect are used, and exposure–response relationships between environmental noise exposure and health outcomes will be calculated. The project will analyze acute BP changes related to short-term aircraft noise exposure (nighttime in particular).

## Materials and Methods

### Study population and study area.

A total of 6,000 persons (men and women, 45–70 years of age) who have lived at least 5 years in the vicinity of the study airports have been selected using existing noise contours around the airports, aiming to maximize exposure contrast. The selection of the study areas was based on existing data on aircraft noise and road traffic noise levels. Recent aircraft noise contours were available for Milano/Malpensa, Berlin/Tegel, Stockholm/Arlanda, London/Heathrow, and Amsterdam/Schiphol. There was limited information for the new Athens airport, but predicted noise contours have been calculated in the planning process. The selection process created exposure contrast to aircraft noise and road traffic noise within countries, ensuring that sufficient numbers of inhabitants in the appropriate age range had expected exposures > 60 dB(A) and < 50 dB(A). The preferred noise distributions, serving as selection guidelines in the HYENA study, are shown in Table 1.

Road traffic noise may be difficult to predict because it is a ground-based source with a complex propagation path from source to receptor. For the initial selection process of the study population, we used local noise data to obtain road traffic exposure classification of locations and populations. If such data were unavailable, two simplified methods derived from more complex models were applied. The first is based on a U.K. method of calculating noise from roads (Great Britain Department of Transport 1988) where exposure depends only on distance to source, speed, and traffic flow. The second is an adaptation of a Dutch method (RMV 2002), which, in addition to the U.K. method, takes into account the numbers of cars, small- and medium-size trucks, and large trucks.

**Figure 1:**
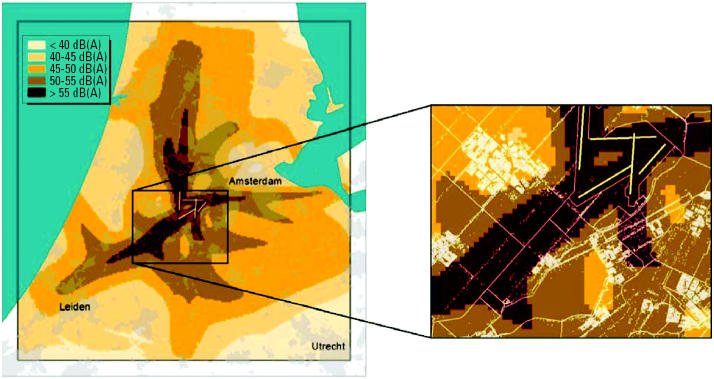
Study area with aircraft noise data at grid level (*L*_den_, 2001) and a close-up showing administrative boundaries and residential address coordinates.

An example of the results of the initial selection in the Netherlands is given in Table 2. Based on the distribution of aircraft noise and road traffic noise in approximately 1 million homes surrounding Schiphol, the numbers of homes available for selection in the various exposure categories are shown in Table 2, which indicates that in the Netherlands it is not possible to comply with the current study demand for 5% of the population to be exposed to levels ≥70 dB(A). However, it is possible to select some additional homes < 70 dB(A), so the requirement for 20% of the population to be exposed to ≥65 dB(A) can be met. The Dutch HYENA study area with the annual average aircraft noise data for 2001 at grid level [day-evening-night noise level (*L*_den_)] and a close-up showing administrative boundaries and residential address coordinates are shown in Figure 1.

In four study areas (Malpensa, Arlanda, Heathrow, and Athens airports), 24-hr BP and continuous nighttime noise will be measured in a subsample (*n* = 100) of highly exposed people (> 65 dB) and referents (*n* = 100; < 50 dB).

### Health outcomes.

#### Blood pressure.

The protocol for assessing BP in HYENA is partly based on protocols previously developed and used in the large multicenter projects INTERSALT (International Study of Electrolyte Excretion and Blood Pressure) and INTERMAP (International Study of Macro-and Micronutrients and Blood Pressure) (Elliott and Stamler 1988).

Until recently the Rz (random zero) sphygmomanometer has been the standard for measuring BP in population studies, but the procedure is prone to errors (Staessen et al. 2000). Automated instruments reduce observer error and can print out measurements, ensuring that recorded data are accurate (O’Brien 2000). These techniques are now well established in clinical research and are increasingly used in occupational and environmental medicine (Staessen et al. 2000). The HYENA study protocol requires that only validated instruments be used. The procedures required for the validation have been thoroughly standardized (O’Brien et al. 2002).

Specially trained staff members (nurse or equivalent) measure BP at three occasions during a single visit. The visits are distributed over the day as far as feasible, to account for diurnal variations in BP. Information about activities of potential influence for BP levels during the assessment day is included in the questionnaire. In a subsample (*n* = 200), 24-hr BP measurements are carried out every 15 min, using validated instruments (Mobilograph; Numed Cardiac Diagnostics, Sheffield, UK).

#### Stress hormones.

A subsample of the study population (*n* = 500) has been identified, stratified by noise level. The participant gathers saliva in a test tube the day before the interview at three occasions; 30 min after awakening, immediately before lunch, and before going to bed. The tubes are collected on the day of the interview. The samples are frozen and kept at a temperature of at least −20°C until analysis. The laboratory determines saliva cortisol using a radioimmunoassay technique and collaborates with a European network for comparisons of saliva cortisol concentrations in different populations.

### Confounders and effect modifiers.

A standardized questionnaire was designed, including validated questions on annoyance and noise disturbance from sources other than air and road traffic (e.g., neighbors) (Fields et al. 2001; Guski et al. 1999). The questionnaire also collects data on major well-known confounders (e.g., dietary habits, smoking, and other lifestyle factors as well as occupational noise exposure) using validated questions from previous studies.

### Noise exposure assessment.

The aim of the noise exposure assessment is to determine individual exposure to aircraft and road traffic noise for each participant, using noise models and noise maps, calculating noise load at grid level. The noise model will calculate required noise levels with a resolution of 1 dB(A). Grid size depends on noise source (the best calculation for aircraft is 250 × 250 m, and for road traffic 10 × 10 m).

Since the start of the HYENA project, much progress has been made in the European Union on the harmonization of noise indicators, calculations, and mapping. Directive 2002/49/EC (European Commission 2002) obliges E.U. members to produce noise maps before 2007 for their larger agglomerations (> 250,000 inhabitants). Therefore, road traffic noise maps will be available for some study areas in the HYENA project. The directive selected *L*_den_ and *L*_Aeq_ 2300–0700 hr (*L*_night_) as the common noise indicators, and methods for noise modeling are described. Current exposure to aircraft and road traffic noise will be calculated for separate periods of the day [*L*_Aeq_ 0700–1900 hr (*L*_day_), L_Aeq_ 1900–2300 hr (*L*_evening_), *L*_night_], which enables the calculation of combinations of the three indicators, including *L*_Aeq,24hr_. These time periods are default values that can be modified according to differences between countries (Directive 2002/49/EC); the day–evening–night level (*L*_den_) is defined by the formula given in Equation 1:


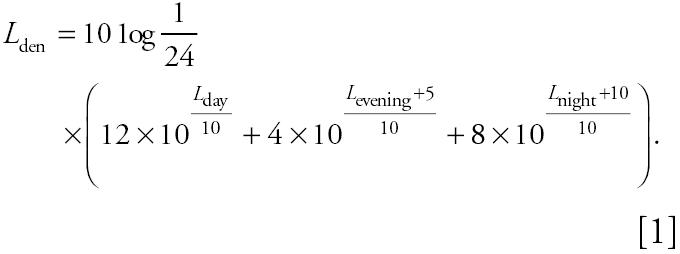


We selected the year 2002 as reference for assessment of current levels of aircraft and road traffic noise. It is assumed that the noise levels in this year are representative of the 5-year time period preceding the health status assessment. If not, teams are allowed to choose another representative year for this period.

Noise exposure will be assessed for each participant by linking home addresses to modeled aircraft and road traffic noise levels. Therefore, knowledge about the exact place of residence of the participants is needed. Address coordinates will be entered in a geographic information system. The difference between noise load at the facade of the sleeping room and that of the living room will be evaluated for road traffic noise. For aircraft noise, this difference is negligible.

Modifiers of individual exposure, such as the orientation of living and bedroom toward roads, window-opening habits, and sound insulation, are assessed during the home visits, using questionnaires and visual inspection. Based on this information, subjects with the same noise level at the front of the house will be stratified according to room orientation. In urban situations, where streets commonly are the predominant noise sources, attenuation of sound levels could be expected between the front and the back of a detached house of (at least) 10 dB(A), and (at least) 20 dB(A) for a terraced house. These attenuation levels will be adapted to each local situation.

Historical exposure to aircraft and road traffic noise over the past 5–10 years will be assessed if feasible. Modeling will not be performed for the years before 2000, but existing noise data will be used for the historical exposure assessment, when available. As a consequence, historical data might be available in local noise indicators only obtained with local noise models. To ensure cross-country comparability, these data will be converted to the noise indicators *L*_den_, *L*_night_, and *L*_Aeq,24hr_. For the conversion, we will model noise levels for the reference year (default 2002) using local models as well as the standard models used in HYENA. Based on a local comparison of model results, historical data will be adjusted to the results of the standard models. Cumulative exposure estimates will be computed by combining the individual (historical) residence noise data with the number of years spent at each address.

To facilitate comparability between the HYENA countries, a common noise model will be used to assess current exposure to aircraft noise. Internationally, the integrated noise model (INM) is at present the most accepted model and will serve as the standard model for assessment of current exposure. For the assessment of aircraft noise, information about airport, aircraft fleet, and runway use is essential, and data on flight tracks and flight procedures need to be collected, as well as meteorologic data and, if applicable, terrain elevation. Flight tracks will be based on radar tracks, representing the actual flown flight tracks. Flight profiles can be based on nominal (International Civil Aviation Organization/INM) profiles. The minimum output of the noise model will be the required noise levels with a resolution of 1 dB(A) on a grid with a minimum resolution of 250 × 250 m. Where feasible, the model estimates will be validated by noise measurements.

Road traffic noise assessment is less uniform among countries. Therefore, locally used models are more tailored to each local situation, emphasizing the collection of good quality data. The European Commission’s Working Group Assessment of Exposure to Noise recently published *The Good Practice Guide for Strategic Noise Mapping* (*GPG*) (European Commission WG-AEN 2003) to facilitate the production and comparability of road noise mapping. The *GPG* contains a tool kit that enables evaluation of the relevant input data with regards to validity and accuracy. HYENA countries will use the tool kits included in the *GPG*.

Countries preferring to use another noise model could use the European interim model on road noise, the French national computation method (CERTU/CSTB/LCPC/SETRA 1997). However, because this model is detailed and complex, HYENA countries may also consider the use of the less complicated models such as the German model RLS-90 (Schade 2004). The *GPG* recommends that the noise model should at least be able to calculate first-order reflections. If necessary, adjustment for reflections could be applied to any (grid-based) noise level to determine noise exposure for people living in dwellings. Although an adjustment is a compromise and will cause some inaccuracies, the *GPG* states that inaccuracies due to data deficiencies are likely to be much more significant.

For the study of short-term effects on BP, noise exposure will be assessed indoors during the night, using a type 1 noise meter (CESVA SC310; CESVA Instruments, SL, Barcelona, Spain), which records noise levels eight times per second, complemented by noise recording, using mp3 players, to allow characterization of the noise source (e.g., aircraft, traffic, indoor).

### Air pollution exposure assessment.

For four airports (Athens, Malpensa, Schiphol, and Heathrow), exposure to air pollution from both aircraft and road traffic will be assessed to explore possible confounding and interactive effects of air pollution on cardiovascular disease. For each of these airports, exposures to traffic-related air pollutants (NO_2_, PM_10_) will be assessed, using results from another E.U.-funded project (Air Pollution Modelling for Support to Policy on Health and Environmental Risks in Europe; APMoSPHERE). Additional modeling will be performed at some airports. Modeled estimates will be validated by monitored data.

In APMoSPHERE, 1-km-resolution emission maps of PM_1 0_, sulfur dioxide, nitrous oxides (NO_x_), and carbon monoxide, by source sector, are being developed for all E.U. countries, using data on source distribution and activity, together with other proxies (e.g., population density) to redistribute national emission totals to local levels. Emissions from most source sectors are modeled as point or area sources, or some combination of the two. Data on point sources are derived from the 2001 European Pollutant Emission Register database (European Commission 2001).

Road transport represents a major source of emission for the pollutants relevant for HYENA (PM_10_, NO_x_). Thus, specific attention needs to be given to estimation of emissions from road transport. Initially, total emissions for each pollutant by vehicle type are obtained, as well as data on the distribution of vehicle types by road type. These are used to subdivide the total emissions by vehicle type into three categories: highways (motorways) and urban and rural roads. Separate calculations are made for urban and rural roads, and the population weights thus computed are used to redistribute the urban and rural emissions across the road network. Maps are integrated to provide a total emission from all vehicle types across all roads.

For airports, data on location and activity are available for each E.U. country. Emissions are estimated on the basis of source area and the level of airport activity. Detailed area data are not available for all airports; therefore, three size classes are applied where these data are lacking: large (25 km^2^) for major international airports, such as Heathrow; medium (9 km^2^) for smaller international airports; and small (1 km^2^) for local and regional airports. Only emissions from aircraft ground movements, take-off, and landing are considered. However, for the airports where detailed modeling will be undertaken (e.g., Heathrow), ground traffic will be included, and we will establish how much this will add to air pollution concentrations. Total national emissions within this sector are thus disaggregated to specific grid squares using activity-weighted areas.

For each participant in the HYENA air pollution substudy (*n* = 4,000), exposure estimates will be made by modeling mean annual and 95th percentile concentrations outdoors at the place of residence. Cumulative air pollution exposure estimates will be computed for each HYENA study participant by integrating place of residence, time spent at each address, and exposure data.

### Study power and statistical analysis.

To detect a mean difference of 3 mmHg systolic BP (140 vs. 143 mmHg, SD = 20 mmHg) between groups in this cross-sectional study, approximately 700 persons are needed per group to achieve 80% power. Similarly, a mean difference of 2 mmHg diastolic BP (85 vs. 87 mmHg, SD = 12 mmHg) can be detected with approximately 600 persons per group. Thus, our study has sufficient power to detect small differences in BP potentially resulting from noise exposure.

We will analyze BP in relation to noise levels in each country and then pool the results in the final analyses using a fixed-effects model. We will analyze the impact of work-related noise by stratifying on occupational noise exposure assessed by the questionnaire. Individuals using antihypertensive drugs will be analyzed separately. Certain indoor environment characteristics (e.g., double glazing) will modify outdoor exposure and will be taken into account by stratified analysis.

## Discussion

Previous studies that have indicated possible associations with airport-related noise and cardiovascular effects most often focused on one airport with relatively small study populations, as noted in an editorial accompanying the study on the Swedish airport (Rosenlund et al. 2001). The editorial suggested that a larger multicenter study was needed to test the hypothesis that airport-generated noise may give rise to raised BP (Pattenden 2001). The HYENA study has taken these suggestions into account as well as combining a large study size with a thorough exposure assessment to achieve maximum resolution in the analysis.

### Selection of study areas.

The selection of study areas involved a number of deliberations to minimize confounding and ensure comparability between countries. Where feasible, we avoided areas where substantial changes in noise exposure had occurred (or would occur) in previous (or coming) years, and we chose areas with suitable noise data, such as road traffic intensity and speed. We aimed to select areas with low migration and to avoid areas with sound insulation programs, where feasible. Finally, we aimed to avoid areas with other sources of noise exposure (e.g., rail, industry) and to choose areas with similar socioeconomic status within countries. Nevertheless, some differences in socioeconomic status between areas are unavoidable and will be controlled for in the analyses.

### Noise modeling.

Although the INM model is the most widely used, some countries may prefer to use their own model, such as the U.K. ANCON (Aircraft Noise Contour model) (Ollerhead 1992). However, the models are very similar, the computation methods being essentially identical, using industry-supplied data to relate aircraft thrust and height with noise emission for individual aircraft types. At London airports, more than one-third of movements are made by aircraft with no matching INM noise power distance data available (Jopson et al. 2002).

The study areas vary among HYENA countries, from urban areas with highly dense populations (Germany and United Kingdom) to mixed (Sweden and the Netherlands) and more rural areas (Greece and Italy). Therefore, there is a marked difference in the complexity of the modeling of road traffic noise, where the more urban areas necessitate a more detailed approach because of complex factors such as attenuation and reflection.

Before the adoption of Directive 2002/49/EC, almost every country in the European Union had its own national noise model. The adaptation of the noise directive will facilitate noise exposure assessment in the HYENA study and contribute to achieving comparable estimates between countries. We are aware that the simplified methods for road traffic noise modeling ignore important modifying factors such as type of road surface, barriers, acceleration near crossing, and surface reflection, but we believe this not to be a major problem for the selection of eligible study participants.

The quality of the noise modeling together with the use of the *GPG* should fulfill the requirements of the HYENA project. However, for home addresses with relatively low road traffic noise exposure, the noise exposure assessment may become inaccurate, because of deviations in the input data despite the use of the *GPG*. For example, traffic intensity might be so low that small deviations from the flows could result in large effects in the calculated noise load. Because input data are critical for the modeling of road traffic noise, HYENA countries will aim for the highest possible accuracy, when selecting the data collection methods according to the tool kits in the *GPG*.

### Air pollution.

Airports generate both air pollution and noise. Aircraft movements are a major source of noise, whereas much of the air pollution (NO_2_, PM_10_) is associated with road transport related to airport activities. As a result, the geographic patterns of noise and air pollution near the airport tend to differ, with noise pollution following the main flight paths at take-off and landing, and air pollution often showing a more regional distribution, determined by the network of feeder roads. These spatial differences in the two pollutants provide the opportunity to examine their independent and interactive effects.

### Exposure misclassification.

As in all epidemiologic studies, the HYENA study will suffer from a degree of exposure misclassification due to uncertainties in modeling noise levels from transport. However, a doubling of traffic will account for an increase of only 3 dB(A). Given an exposure range of about 45–75 dB(A), the impact on the analyses will probably not be substantial. Nevertheless, it is clear that the rather complex modeling involved will give rise to uncertainty, which we will assess by performing sensitivity analyses in the final data analysis.

### Blood pressure measurements.

Single measurements or multiple readings taken by an observer at one or even several times through the day may reflect a subject’s true BP only to a minor extent (Staessen et al. 2000). Nevertheless, this method has commonly been used in environmental epidemiology. Ambulatory BP monitoring makes it possible to record the BP throughout the whole day in patients engaged in their normal activities and provides a reliable estimate of their BP over a 24-hr period. However, although 24-hr monitoring is preferable, the costs are usually prohibitive when the study population is large.

### Stress hormones.

Three aspects of saliva cortisol assessments are crucial in relation to reactions to long-lasting stressors such as aircraft noise. First, as long as subjects have retained ability to up-regulate cortisol, levels may become elevated. This may be particularly relevant during the early morning hours. Second, when the life situation has been disturbed for a long time, the ability to down-regulate cortisol may be inhibited. This is particularly relevant during the late hours when cortisol excretion in normal subjects is much lower than during the early hours immediately after awakening. Finally, it is believed that subjects who have suffered from severe stressors for a long time may have exhausted the ability of the cortisol system to regulate in the normal way. In such cases, levels become abnormally low and show very little variation. Saliva cortisol measurements may show high and low levels, and high as well as low variability during exposure to long-lasting stressors. The exhausted group, however, is small in most studies of normal populations. Therefore, long-lasting stressor exposure may result in elevated cortisol levels, and perhaps lowered ability to decrease levels at night before bedtime.

Saliva sampling has the advantage above collection of blood specimens in that it is easy and cheap to administer. The study subjects can easily be instructed to collect samples themselves. Hence, many samples can be collected, and this makes it possible to study circadian disturbances in cortisol regulation.

### Short-term effects of night noise and BP dipping.

Numerous laboratory studies have investigated sleep disturbances by acute noise; the effects on BP have been rarely assessed, and only a few field studies have been published (Babisch 2002). We will study short-term effects of noise on BP in 200 subjects, primarily to explore a possible link between short- and long-term effects through the inhibition of nighttime BP dipping, which is an established risk factor for cardiovascular disease (Lee et al. 2005; Ohkubo et al. 2002). We will compare day and night average BP levels and examine the association of nighttime BP to the average noise levels in the bedroom, assessing effects of aircraft noise and other noise sources separately.

## Conclusion

To our knowledge, the HYENA study is the first large multicenter study designed to assess the effects of exposure to aircraft and road traffic noise on BP and cardiovascular disease, as well as possible modifying effects of exposure to air pollution. Study airports are located in several different European countries offering a wide range of noise exposure, ensuring exposure contrast and cross-country variations (e.g., due to cultural and climate differences), which will be addressed in the analysis. Final results can be expected in 2007.

## Figures and Tables

**Table 1:** Preferred distributions for aircraft noise and (in parentheses) for road traffic noise.

Cumulative fraction	Noise level [dB(A)]
0.05 of the population exposed above	70
0.20 (0.25) of the population exposed above	65
0.35 (0.40) of the population exposed above	60
0.45 of the population exposed above	55
0.15 of the population for aircraft and road traffic noise exposed below	51

**Table 2:** Air and road traffic noise levels at the facade of houses in an area of 55 × 55 km near Schiphol Airport in Amsterdam.

	*L*_den_ aircraft noise [dB(A)]							
*L*_Aeq_07–23 road noise [dB(A)]	≤ 50	51–55	56–60	61–65	66–70	≥ 71	Total	Percent
≤ 45	126,000	19,200	3,750	550	40	0	149,540	12.71
46–50	204,000	31,900	4,800	650	20	0	241,370	20.52
51–55	253,000	44,700	5,800	1,450	200	20	305,170	25.94
56–60	330,000	22,000	2,200	1,600	70	10	355,880	30.25
61–65	98,000	9,100	750	400	40	10	108,300	9.21
66–70	11,000	1,600	150	100	30	10	12,890	1.10
≥ 71	2,800	250	30	100	0	0	3,180	0.27
Total	1,024,800	128,750	17,480	4,850	400	50	1,176,330	100.00
Percent	87.12	10.95	1.49	0.41	0.03	0.00	100.00	

*L*_Aeq_07–23, A-weighted average sound pressure level [dB(A)], day and evening.
